# Targeted Inhibition of GATA-3 by Pyrrothiogatain: Implications for Adipocyte Biology and Inflammatory Response

**DOI:** 10.3390/cells14020100

**Published:** 2025-01-10

**Authors:** Shamma Almuraikhy, Maha Alser, Khaled Naja, Aisha Al-Malki, Nayef A. Mazloum, Mohamed A. Elrayess

**Affiliations:** 1 Biomedical Research Center, Qatar University, Doha P.O. Box 2713, Qatar; salmuraikhy@qu.edu.qa (S.A.); maha.alser@qu.edu.qa (M.A.); khaled.naja@qu.edu.qa (K.N.); 2 Department of Microbiology and Immunology, Weill Cornell Medicine-Qatar (WCM-Q), Qatar Foundation, Doha P.O. Box 24144, Qatar; aym2003@qatar-med.cornell.edu (A.A.-M.); nam2016@qatar-med.cornell.edu (N.A.M.); 3 College of Medicine, QU Health, Qatar University, Doha P.O. Box 2713, Qatar

**Keywords:** GATA-3, Pyrrothiogatain, adipogenesis, inflammation, oxidative stress

## Abstract

GATA-3 is a master regulator of preadipocyte differentiation and function. Pharmacological or genetic targeting of GATA-3 will allow us to understand the function of GATA-3 in regulating metabolism, insulin signaling, and inflammation. Pyrrothiogatain, a novel small molecule inhibitor of GATA family proteins, has emerged as a promising tool for modulating GATA-3 activity. This study aims to investigate the specificity of Pyrrothiogatain in regulating GATA-3-mediated preadipocyte differentiation and adipokine secretion under normal and pathological conditions. Wild-type and GATA-3 knockout 3T3-L1 cells were treated with different concentrations of Pyrrothiogatain in the presence and absence of 4-hydroxy-2-nonenal (4HNE), an inducer of oxidative stress and impairment of adipogenesis. As expected, GATA-3 knockout cells exhibited enhanced adipogenic capacity, characterized by increased cell and lipid droplet sizes, and upregulated expression of key adipogenic markers including CEBPβ, PPARγ, and PGC-1α. Pyrrothiogatain treatment reduced cell proliferation in both wild-type and GATA-3 knockout 3T3-L1 cells, but did not alter their adipogenic capacity. Furthermore, Pyrrothiogatain lowered secreted IL-6 levels and attenuated 4-HNE-induced TNF-α elevation in wild-type, but not in GATA-3 knockout cells. Co-treatment of 4-HNE and Pyrrothiogatain led to increased cell size, suggesting complex interactions between oxidative stress and GATA protein inhibition. This effect was similar to GATA-3 knockout cells, indicating Pyrrothiogatain’s potential to modulate cellular stress responses independently of GATA-3 inhibition. These results reveal that Pyrrothiogatain’s effects on adipocyte biology extend beyond simple GATA-3 inhibition. While GATA-3 knockout primarily affects adipogenesis, Pyrrothiogatain modulates inflammatory responses and potentially cellular stress mechanisms without directly impacting adipocyte differentiation. This study provides new insights into the multifaceted actions of Pyrrothiogatain and highlights its potential as a therapeutic agent for lowering inflammation and oxidative-stress-related aspects of metabolic disorders, distinct from the direct modulation of adipogenesis.

## 1. Introduction

Adipogenesis, the process by which preadipocytes differentiate into mature adipocytes, is regulated by a complex gene expression program involving key regulators such as peroxisome proliferator-activated receptor γ (PPARγ) and CCAAT/enhancer-binding proteins (C/EBPs) [1]. The anti-adipogenic transcription factor GATA-3 has been identified as one of the key molecular targets responsible for the impairment of adipogenesis. GATA-3 expression has been observed in primary preadipocytes from white adipose tissue (WAT) and in preadipocyte cell lines such as 3T3-L1 [2,3,4,5]. The expression of GATA-3 is higher in insulin-resistant obese individuals compared to BMI-matched insulin-sensitive counterparts [4,6]. Studies have revealed a potential link between GATA-3, interleukin-6 (IL-6), and impaired subcutaneous adipogenesis in insulin-resistant individuals [4]. Inhibition of GATA-3 can enhance adipogenic capacity in human primary preadipocytes [6], reverse 4-hydroxynonenal (4HNE)-impaired adipogenesis, and induce changes in insulin signaling in 3T3-L1 preadipocytes [5]. These findings suggest that GATA-3 inhibition could be a potential therapeutic strategy for improving adipogenesis and insulin sensitivity [6,7]. The suppression of GATA-3 has also shown promising results in reducing inflammation and reversing insulin resistance in vivo [7]. This growing body of evidence highlights the potential of GATA-3 as a therapeutic target for obesity-related metabolic disorders, including insulin resistance and type 2 diabetes.

Various GATA-3 inhibitors have been studied for their potential modulation of adipogenesis, inflammation, and cellular stress responses. Pyrrothiogatain (3-(2,5-dimethyl-1H-pyrrol-1-yl) thiophene-2-carboxylic acid) was identified as a novel inhibitor of GATA-3 DNA-binding activity [8]. It inhibits the DNA-binding activity of GATA-3 as well as other members of the GATA protein family, including GATA2, GATA4, and GATA5 [8]. It also inhibits the interaction between GATA-3 and SOX4, suggesting it interacts with the DNA-binding region of GATA-3 [8]. As an inhibitor of GATA family proteins, Pyrrothiogatain can also suppress Th2 cell differentiation without impairing Th1 cell differentiation [8], leading to a lower expression and production of Th2 cytokines like IL-4, IL-5, and IL-13 [8]. Therefore, the use of Pyrrothiogatain could provide a new therapeutic tool for studying GATA family protein function and regulating Th2 cytokine production [8]. This could be particularly relevant for treating conditions associated with excessive Th2 cytokine production, such as type-2 allergic inflammation, as well as conditions characterized by impaired adipogenesis and adipokine release, such as insulin resistance and type 2 diabetes.

This study aims to investigate the impact of Pyrrothiogatain on adipocyte development and function using both GATA-3 wild-type and knockout 3T3-L1 cells. It seeks to determine the specificity of Pyrrothiogatain’s effects through GATA-3 inhibition and to explore its role in regulating GATA-3-mediated preadipocyte differentiation and adipokine secretion under both normal and pathological conditions.

## 2. Materials and Methods

### 2.1. 3T3L-1 Cell Culture and Maintenance

Mouse 3T3L-1 preadipocytes (Zen-Bio, Durham, NC, USA) were cultured in Dulbecco’s modified Eagle medium (DMEM) (Invitrogen, Paisley, Scotland), supplemented with 10% calf serum (Invitrogen) and 1% penicillin/streptomycin antibiotics (Invitrogen). The cells were maintained in a 37 °C humidified incubator under 5% CO_2_ and were sub-cultured after reaching 70–80% confluency.

### 2.2. GATA-3 Knockout Cell-Line Generation

The lentiviral system using Guide RNA lentiCRISPR v2 constructs targeting GATA-3 and non-targeting (NT) control were purchased from (Genscript, Nanjing, China) and were used to generate the GATA-3 knockout 3T3L-1 cell line. The GATA-3 gRNA target sequences were: GATA-3 gRNA-1: CCGGGTTCGGATGTAAGTCG and NT control gRNA: GCTTTCACGGAGGTTCGACG. To generate lentiviral particles, human embryonic kidney 293 (HEK293T) cells (ATCC) were cultured in high glucose DMEM media supplemented with 10% fetal bovine serum (FBS) (AlphaFBS-HI, Alphabioregen, Boston, MA, USA) and 1% penicillin/streptomycin antibiotics (Invitrogen) [9,10]. The cells were maintained and sub-cultured at 70–80% confluence. HEK293 cells were co-transfected with lentivirus packaging plasmids (pMD2.G and psPAX2) alongside plasmids targeting GATA-3 or control non-targeting sequence (NT) and incubated for 6 h using lipofectamine 2000 following the manufacturer’s instructions. Then, the transfection media was switched to complete media (DMEM + 10% FBS) and incubated for 72 h. The lentivirus-containing supernatants were then collected, centrifuged at 1500 RPM, 4 °C for 5 min filtered through 0.45 μm membrane (Millipore, Burlington, MA, USA), and used or stored at −80 °C for later use. Early passage cultured 3T3-L1 preadipocytes were transduced with lentiviral particles with 4 µg/mL polybrene (Santacruz Biotechnology, Dallas, TX, USA) for 48 h, sub-cultured under puromycin selection (2 µg/mL) 3~4 times as previously described [9,10]. GATA-3 knockout was then validated using Western blotting using antibodies for GATA-3 and GAPDH as a loading control.

### 2.3. Adipocyte Differentiation

The cells were seeded in 6-well plates in triplicate with a density of 200,000 cells/well in DMEM media and cultured to 100% confluence. After two days (D0, over-confluence state), the cells were induced to differentiate using differential media containing 500 μM 3-isobutyl-1-methylxanthine (IBMX) (Sigma, Saint Louis, MO, USA), 1 μM dexamethasone (Sigma), and 10 μg/mL insulin (Sigma). The media were supplemented with 10% FBS and 1% penicillin/streptomycin for an additional two days. Then, at D2, the media were shifted to post-differentiation media containing 10 μg/mL insulin and supplemented with 10% FBS and 1% antibiotic. The media were changed every two days for the rest of the experiment (until D10). Cells were collected for RNA and protein extraction at different time points through the experiment: Days 0, 1, 2, (early differentiation), 4, 6, 8, and 10 (late differentiation) for further processing. For knockout cells, the same protocol was followed while supplementing the media with 2 µg/mL of puromycin for continuous selection.

### 2.4. Oil Red O Staining

Differentiated cells were stained with Oil Red O (ORO) at the end of the differentiation experiment (D10) to assess the adipogenic capacity following the standard protocol. Briefly, the cells were washed with phosphate-buffered saline (PBS) and fixed with 4% paraformaldehyde (PFA) for 15 min at RT. After rinsing with PBS, the cells were washed with 60% isopropanol in PBS for 1 min. Subsequently, the cells were incubated in 60% ORO stain (Sigma-Aldrich, Saint Louis, MO, USA) in PBS for 1 h at RT. After staining, the cells were washed in 60% isopropanol followed by water and mounted in 70% glycerol. Stained cells were examined under a digital microscope (EVOS™, Eindhoven, The Netherlands).

### 2.5. Protein Extraction and Western Blotting

Western blotting was conducted to confirm the knockout experiments and to assess the expression level of certain proteins at the protein level following the standard protocol. Briefly, on ice, cells were washed with cold PBS and lysed with RIPA lysis buffer (ThermoFisher Scientific, Waltham, MA, USA) supplemented with protease inhibitor and phosphatase inhibitor (Sigma). Protein lysates were then homogenized by sonication and centrifuged at 15,000× *g*, 4 °C. Protein-containing supernatants were collected, and concentrations were estimated using DC Protein Assay (Bio-Rad, Hercules, CA, USA) on a CLARIOstar plate reader. Then, 30 μg of whole protein were loaded and separated through SDS-PAGE with a molecular marker (ThermoFisher Scientific), blotted on PVDF membrane. The membranes were blocked with 4% bovine serum albumin (BSA) in Tris-buffered saline (TBS), washed with TBS containing 0.1% Tween-20 (TBST), and probed with the antibodies of interest using the manufacturer’s recommended concentrations in 2% BSA at 4 °C overnight on a rocker. The excess antibody was then washed 3× with TBST, then the blots were incubated with the matching HRP-conjugated secondary antibodies at RT for 1 h, washed 3× with TBST, and treated with SuperSignalTM West Dura Extended Duration Substrate (ThermoFisher Scientific) for 1 min before detection. The ChemiDoc MP imaging device (Bio-rad) was used to detect and image the blots. The antibodies used in this study are GATA-3 (Abcam 199428, UK), GAPDH (Cell signaling Technology 2118, Danvers, MA, USA), β-ACTIN (Cell signaling Technology 3700, USA), PPARγ (Cell signaling Technology 2443, USA), CEBPα (Cell signaling Technology 2295, USA), and CEBPβ (Abcam 32358, Cambridge, UK). The secondary antibodies used are anti-mouse IgG (Cell signaling Technology 7076, USA) and anti-rabbit IgG (Cell signaling Technology 7074, USA). Protein bands were quantified using the ImageJ software (Version 1.54k) and normalized to the loading control intensity (GAPDH or β-actin) of the same sample and to the control sample.

### 2.6. Assessment of Gene Expression Using RT-PCR

The total RNA was extracted from the cells using the Qiazol lysis buffer (QIAGEN, Venlo, The Netherlands) and further processed using the miRNeasy Mini Kit (QIAGEN, Venlo, The Netherlands) following the manufacturer’s suggestion to purify RNA. A DNA digestion step was added by incubating the sample with DNase I (QIAGEN) for 15 min before RNA elution. RNA quantity and quality were measured on a nanodrop spectrophotometer. Then, 1 ug of template RNA was reverse transcribed to cDNA using the High-Capacity RNA-to-cDNA Kit (Applied Biosystems, Foster City, CA, USA). The product cDNA was used in the following RT-PCR to assess the levels of selected genes at the RNA levels. Briefly, SYBR green master mix (Applied Biosystems) was used with specific primers and cDNA samples in triplicate and run using the QuantStudio™ 6 Flex Real-Time PCR System (Applied Biosystems) according to the manufacturer’s suggestion. The machine was set to the following settings: denaturation (50 °C, 20 s; 95 °C, 10 min, 40 amplification cycles (95 °C, 15 s; 60 °C, 1 min) and melting curve analysis steps (95 °C, 15 s; 60 °C, 1 min; 95 °C, 30 s; 60 °C, 15 s). The gene expression was calculated from the CT values by the end of the experiment, normalized to the internal control (NONO) and quantified by the comparative threshold cycle (ΔΔCT) method. The primers used for gene expression analysis are as follows: GATA-3 (forward: GAACCGGCCCCTTATCAAG, reverse: ACAGTTCGCGCAGGATGTC) and PPAR-γ (forward: ATTGAGTGCCGAGTCTGTGG, reverse: GCCCAAACCTGATGGCATTG).

### 2.7. Secreted Cytokine Profiling

Profiling of secreted cytokines from differentiated NT and GATA-3 knockout cells was performed using media supernatants collected following the completion of differentiation. Accumulated levels of secreted IL-6 and TNF alpha in the last 4 days before staining were measured using Inflammatory Cytokine mouse Magnetic 6-Plex (Life Technologies, Carlsbad, CA, USA) according to the manufacturer’s instructions and assessed by Luminex Flexmap 3D using xPONENT 4.2 software (Luminex, Madison, WI, USA).

### 2.8. Statistical Analysis

The statistical analysis was conducted using Student’s *t*-test or ordinary one-way ANOVA (Followed by Dunnett’s post hoc test) using GraphPad Prism software (Version 10.2.3). Statistical significance was defined as *p* ≤ 0.05. Data are presented as mean ± standard error of the mean (SEM).

## 3. Results

### 3.1. GATA-3 Knockout Improves Adipogenic Capacity

**Figure 1:**
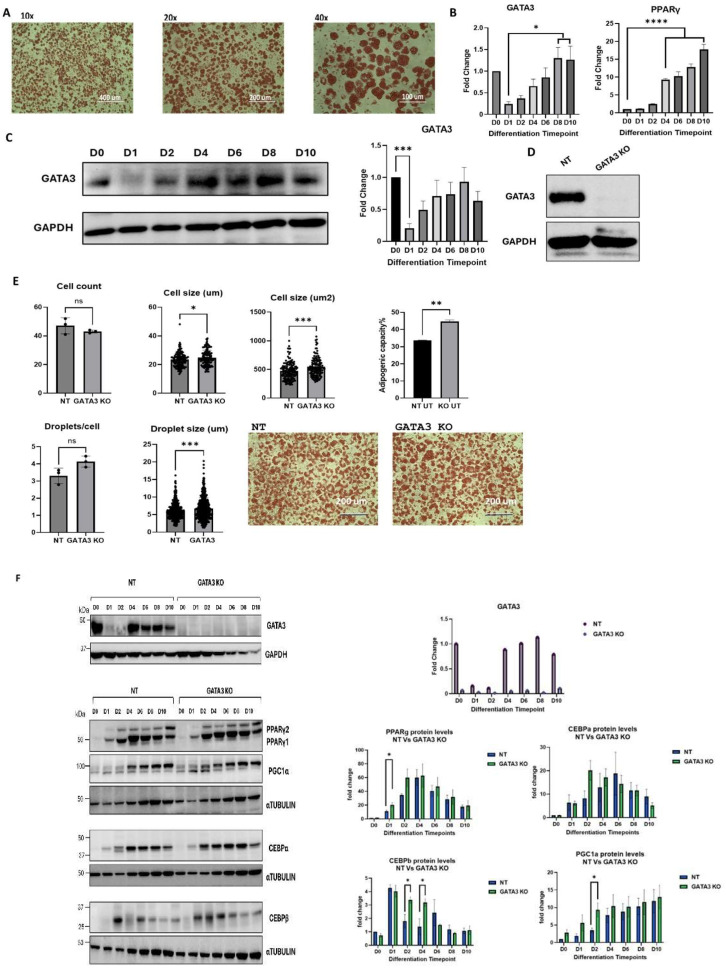
(**A**) Oil Red O staining of 3T3-L1 cells at the end of differentiation (10×, 20×, and 40× magnification). (**B**) Expression levels of GATA-3 and PPARγ mRNA at various differentiation time points for 3T3-L1 cells. (**C**) Expression levels of GATA-3 protein in 3T3-L1 cells at different differentiation time points. Levels were measured by Western blotting. (**D**) GATA-3 knockout validation at the protein level (Western blotting). (**E**) Comparison of the differentiation capacity between GATA-3 knockout (KO) 3T3L-1 cells and non-targeting (NT) control cells. Cells were fixed and stained with Oil Red O on day 10 (final differentiation). Three images were captured per treatment, and four fields per image were quantified using Image J (USA). (**F**) Expression levels of PPARγ, CEBPα, CEBPβ, and PGC-1α by Western blotting and RT-PCR at various differentiation time points in NT and GATA-3 KO cells. The expression levels were normalized to GAPDH and compared to NT D0 for RT-PCR to calculate the fold changes presented. Levels were measured by Western blotting in three independent experiments, normalized to α-TUBULIN, and compared to NT D0 to calculate fold changes. The fold changes for each time point were analyzed using ordinary one-way ANOVA on GraphPad Prism. * = *p* < 0.05 (n = 3). ns: non-significant. * *p* < 0.05, ** *p* < 0.01, *** *p* < 0.001, **** *p*< 0.0001.

The differentiation of 3T3-L1 cells into adipocytes was investigated by ORO staining (Figure 1A), and the levels of GATA-3 and PPARγ were measured using RT-PCR and Western blotting at various time points (Figure 1B,C). As expected, upon induction of differentiation, a sharp drop in GATA-3 expression was observed, which was associated with a gradual increase in PPARγ expression. Following the completion of differentiation, GATA-3 expression returned to baseline levels, while PPARγ expression remained elevated (Figure 1B,C). A lentivirus system was used to knock out GATA-3 in 3T3-L1 cells and confirmed by Western blotting analysis (Figure 1D). GATA-3 knockout was found to enhance adipogenesis following the completion of the differentiation protocol, as evidenced by increased ORO staining, which was associated with larger cell and lipid droplet size (Figure 1E). Additionally, the expression of adipogenesis markers (CEBPβ, CEBPα, and PPARγ) and the mitochondrial marker (PGC-1α) was upregulated in GATA-3 knockout cells (Figure 1F).

### 3.2. Pyrrothiogatain Effect on Proliferation and Differentiation of 3T3L1 Cells

**Figure 2:**
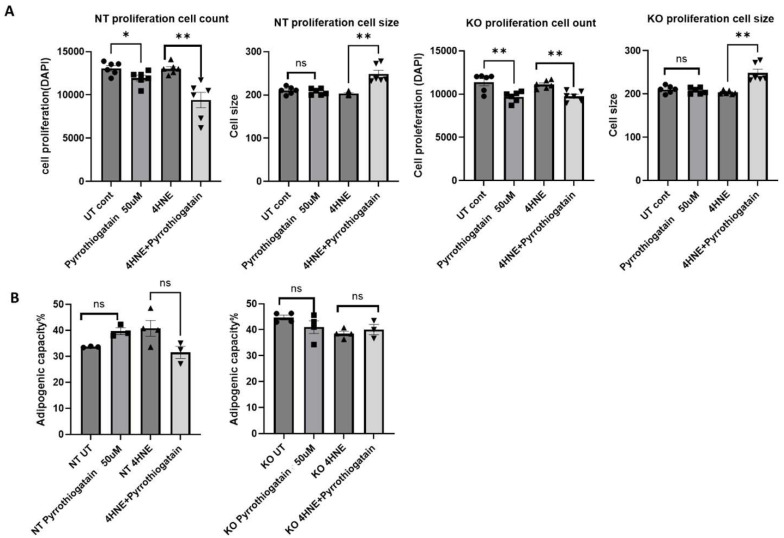
(**A**) The effect of Pyrrothiogatain on cell proliferation and size following 4-day treatment period. (**B**) Adipogenic capacity (%) following treatment with Pyrrothiogatain and 4-HNE in non-targeting (NT) and GATA-3 knockout (KO) cells, including untreated (UT) controls. Data are presented as mean ± SEM, with statistical differences determined using a *t*-test (n = 4). ns: non-significant. * *p* < 0.05, ** *p* < 0.01.

Treatment of 3T3L1 cells with 50 μM Pyrrothiogatain for 4 days led to a decrease in cell count in both NT and GATA-3 knockout cells. Interestingly, when combined with 4-HNE, Pyrrothiogatain treatment resulted in an increase in cell size for both cell types (Figure 2A). However, Pyrrothiogatain did not significantly affect adipogenic capacity (Figure 2B).

### 3.3. Pyrrothiogatain Reduces Inflammation in 3T3L-1 Preadipocytes

**Figure 3:**
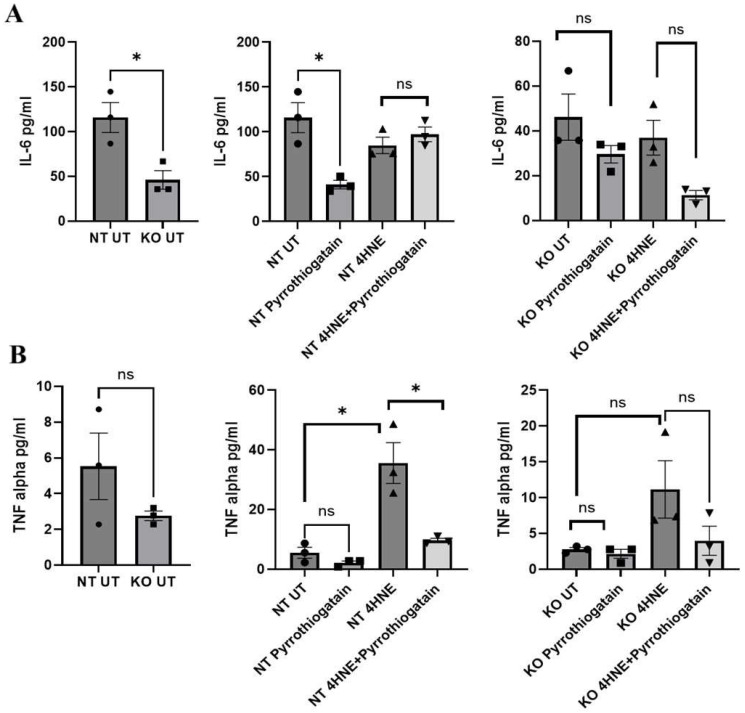
Secreted cytokine levels of IL-6 (**A**) and TNF-alpha (**B**) at baseline and after treatment with Pyrrothiogatain and 4-HNE in non-targeting (NT) and GATA-3 knockout (KO) cells, including untreated (UT) samples. Data presented as mean ± SEM, and the difference was calculated using *t*-test (n = 3). ns: non-significant. * *p* < 0.05.

As shown in Figure 3, the baseline secreted cytokine levels from 3T3L-1 showed no significant differences between NT and GATA-3 knockout cells. However, treatment of 3T3L-1 with 50uM of Pyrrothiogatain in NT cells lowered secreted IL-6 levels, suggesting an anti-inflammatory effect. This effect was not observed in GATA-3 knockout cells, indicating that GATA-3 may be involved in the IL-6 regulatory pathway affected by Pyrrothiogatain. In NT cells, 4-HNE treatment increased secreted TNF-α levels, consistent with its known pro-inflammatory effects. Subsequent treatment with Pyrrothiogatain significantly reduced TNF-α levels. This observation further supports the anti-inflammatory potential of Pyrrothiogatain, particularly in the context of oxidative-stress-induced inflammation.

## 4. Discussion

The therapeutic approach of targeting GATA-3 for inflammatory diseases has been under investigation for the last decade using antisense technologies, such as DNAzymes [11]. Small molecule inhibitors like Pyrrothiogatain have been developed to target the DNA-binding activity of GATA family members, including GATA-3 [11]. The emerging data provide valuable insights into the role of GATA-3 in adipogenesis and the effects of Pyrrothiogatain as a small molecule inhibitor of GATA family proteins on adipocyte function and inflammatory markers. Validating GATA-3 as a potential therapeutic target would be a crucial first step in designing future first in class therapy for improving adipose tissue adipogenesis and restoring insulin sensitivity.

Our study demonstrated that knocking out GATA-3 using a lentiviral system enhances adipogenic capacity by increasing cell and lipid droplet sizes. This enhancement is characterized by the upregulated expression of CEBPβ, PPARγ, and PGC-1α. These findings align with a previous study showing that targeting GATA-3 expression with DNAzyme induced adipogenesis in primary human preadipocytes, resulting in more mature adipocytes compared to untreated cells. This study demonstrated the role of GATA-3 as a negative regulator of adipogenesis across species [7]. Another study found that Creb3l4 knockdown in 3T3-L1 preadipocytes resulted in increased expression of PPARγ2 and C/EBPα, partly due to decreased expression of GATA-3 [12]. This suggests a negative correlation between adipogenic markers and GATA-3, reinforcing its role as an inhibitor of adipogenesis. The research also indicates that inhibiting GATA-3 improved adipocyte differentiation and rescued insulin sensitivity in insulin-resistant cells [5]. One study found that the upregulation of the anti-adipogenic gene GATA-3 in insulin-resistant cells was associated with reduced adipogenic capacity compared to those from insulin-sensitive individuals [4]. These studies suggest that GATA-3 suppresses the transition from preadipocytes to adipocytes by inhibiting the expression and activity of PPARγ2 and C/EBPs [3,13]. Additionally, GATA-3 directly interacts with and binds to C/EBPα and C/EBPβ, which is essential for its negative regulation of adipogenesis [13].

Pyrrothiogatain is a novel inhibitor of GATA-3 DNA-binding activity [14]. This study is the first to report the effects of Pyrrothiogatain on 3T3-L1 cell proliferation and differentiation. Our results showed that Pyrrothiogatain reduced cell proliferation in both wild-type and GATA-3 knockout 3T3-L1 cells, with no significant impact on adipogenic capacity. The observed reduction in cell proliferation highlights Pyrrothiogatain’s potential as an inhibitor of cellular growth.

The lack of significant improvement in adipogenesis after cell treatment with Pyrrothiogatain, unlike cell GATA-3 knockout, could be attributed to several factors. Pyrrothiogatain acts as an inhibitor of GATA family proteins, particularly GATA-3, by suppressing their DNA-binding activity. However, this inhibition may not be as complete or permanent as a genetic knockout. Additionally, it may not affect other mechanisms through which GATA factors regulate adipogenesis [2]. Cells treated with Pyrrothiogatain may activate compensatory pathways to maintain GATA function or support other anti-adipogenic processes, a response that may not occur in genetic knockout models. The impact of Pyrrothiogatain on adipogenesis could also depend on the timing, duration, and dosage of treatment. Furthermore, Pyrrothiogatain may exhibit off-target effects on other proteins or influence additional cellular processes, which could indirectly affect adipogenesis and counteract its GATA-3-inhibitory effects.

In non-targeted cells, Pyrrothiogatain treatment decreased IL-6 levels, indicating its anti-inflammatory potential. This effect was not observed in GATA-3 knockout cells, where IL-6 levels were already reduced due to the absence of GATA-3. These findings suggest that both Pyrrothiogatain treatment and GATA-3 knockout play similar roles in reducing IL-6. A recent study found that GATA-3 inhibition in 3T3-L1 cells enhanced adipocyte differentiation, reduced inflammation, and improved insulin sensitivity [5]. Our findings suggest that GATA-3 may play a role in the IL-6 regulatory pathway affected by Pyrrothiogatain and highlight its potential role as an anti-inflammatory target.

Our findings also showed that exposure to 4-HNE, a reactive aldehyde known to increase inflammation, oxidative stress, and insulin resistance in preadipocytes [15], increased TNF-α levels in non-targeted, but not in GATA-3 knockout, cells. As a lipid peroxidation product, 4-HNE appears to activate inflammatory pathways that enhance TNF-α production [16]. The lack of this response in GATA-3 knockout cells indicates that GATA-3 is a crucial mediator of this inflammatory process. The previous research supports that 4-HNE regulates TNF-α gene transcription through indirect mechanisms [17]. In human adipocytes, for instance, 4-HNE promotes TNF-α gene transcription through the inhibition of the miR-29b-SP1-TNF-α pathway and promotion of the ETS1-TNF-α pathway [17]. 4-HNE induces inflammatory responses; including increasing COX-2 expression via p38MAPK activation [16,18]. Suppressing GATA-3 in 3T3-L1 mouse preadipocytes has been shown to reverse impaired adipogenesis and alter the expression of inflammatory cytokines, including IL-6 [5]. Our results suggest that GATA-3 may also play a role in regulating TNF-α production in response to oxidative stress.

Interestingly, Pyrrothiogatain treatment significantly reduced the TNF-α elevation caused by 4-HNE in non-targeted cells, thereby attenuating its associated inflammatory effects. This reduction was observed only in 3T3-L1 cells and not in GATA-3 knockout cells, where TNF-α elevation was absent. This result suggests that Pyrrothiogatain’s primary mechanism of action involves GATA-3 inhibition. The absence of this effect in GATA-3 knockout cells confirms that Pyrrothiogatain’s impact on TNF-α is mediated through GATA-3. While these results strongly implicate GATA-3, further studies are needed to determine whether Pyrrothiogatain also modulates other pathways involved in the inflammatory response to oxidative stress.

The co-treatment of 4-HNE and Pyrrothiogatain led to increased cell size in both NT and GATA-3 knockout cells, indicating that Pyrrothiogatain may modulate cellular stress responses through mechanisms independent of GATA-3 inhibition. Pyrrothiogatain may affect other transcription factors or signaling pathways involved in adipocyte differentiation and size regulation. Pyrrothiogatain inhibits the DNA-binding activity of GATA-3 and other GATA family proteins, suggesting it may affect multiple transcription factors involved in adipogenesis regulation [8].

We acknowledge some limitations in our study. The study was conducted using 3T3-L1 cells, which may not fully represent the complexity of human adipose tissue. Future research should validate these findings and assess their physiological relevance in primary human adipocytes and in vivo models. Additionally, the observed effects of Pyrrothiogatain on cell size and stress responses may be confounded by its potential cytotoxicity or off-target effects on cellular signaling pathways. Future studies are warranted to include a deeper investigation into the molecular pathways underlying Pyrrothiogatain’s effects on inflammation and cellular stress.

## 5. Conclusions

Pyrrothiogatain represents a novel approach for targeting the action of GATA-3 in preadipocytes by reducing adipokine secretion, particularly via modulating IL-6 and TNF-α levels, without affecting preadipocyte differentiation. Moreover, the interplay between 4-HNE-induced oxidative stress and Pyrrothiogatain’s anti-inflammatory effects provides an interesting model for studying the relationship between oxidative stress and inflammation in adipocytes. This study provides new insights into the potential therapeutic effect of Pyrrothiogatain through reducing inflammation and oxidative-stress-related aspects of metabolic disorders, distinct from direct modulation of adipogenesis. Further studies are needed to elucidate its molecular targets and to explore its implications in adipose tissue development and metabolic regulation.

## Data Availability

Data are available from the corresponding author upon reasonable request.
